# The Self-esteem Stability Scale (SESS) for Cross-Sectional Direct Assessment of Self-esteem Stability

**DOI:** 10.3389/fpsyg.2018.00091

**Published:** 2018-02-13

**Authors:** Tobias Altmann, Marcus Roth

**Affiliations:** Institute of Psychology, University of Duisburg-Essen, Essen, Germany

**Keywords:** self-esteem stability, scale development, questionnaire, validity, direct assessment

## Abstract

Self-esteem stability describes fluctuations in the level of self-esteem experienced by individuals over a brief period of time. In recent decades, self-esteem stability has repeatedly been shown to be an important variable affecting psychological functioning. However, measures of self-esteem stability are few and lacking in validity. In this paper, we present the Self-Esteem Stability Scale (SESS), a unidimensional and very brief scale to directly assess self-esteem stability. In four studies (total *N* = 826), we describe the development of the SESS and present evidence for its validity with respect to individual outcomes (life satisfaction, neuroticism, and vulnerable narcissism) and dyadic outcomes (relationship satisfaction in self- and partner ratings) through direct comparisons with existing measures. The new SESS proved to be a stronger predictor than the existing scales and had incremental validity over and above self-esteem level. The results also showed that all cross-sectional measures of self-esteem stability were only moderately associated with variability in self-esteem levels assessed longitudinally with multiple administrations of the Rosenberg Self-Esteem Scale. We discuss this validity issue, arguing that direct and indirect assessment approaches measure relevant, yet different aspects of self-esteem stability.

## Introduction

The majority of self-esteem research has focused on the global level of self-esteem (i.e., “the individual's positive or negative attitudes toward the self as a totality”; (Rosenberg et al., 1995), p. 141). According to Rosenberg (1979), an individual with a high level of self-esteem can be characterized as follows: “He has self-respect, considers himself a person of worth. Appreciating his own merits, he nonetheless recognizes his faults […]. The term ‘low self-esteem’ […] means that the individual lacks respect for himself, considers himself unworthy, inadequate, or otherwise seriously deficient as a person” (p. 54; this description can be assumed to be true for all sexes). This statement shows that self-esteem is conceptualized more or less as an individual trait, with day-to-day fluctuations in feelings of self-worth dismissed as measurement error. However, a growing number of studies in recent decades have expanded the meaning of self-esteem by differentiating between the global level of self-esteem in general and self-esteem stability.

Self-esteem stability has been defined as the extent to which an individual experiencesshort-termfluctuations in self-esteem (e.g., Kernis, 2005). Even though substantial correlations are usually found between self-esteem stability and self-esteem level (see Okada, 2010), recent studies have consistently found an incremental validity of self-esteem stability over and above self-esteem level in predicting variables relevant for psychological adjustment or functioning. In general, research has shown that a higher degree of self-esteem stability is associated with better adjustment or functioning. This is true both for individual concepts such as neuroticism/emotional stability (Butler et al., 1994), depression (Kim and Cicchetti, 2009), and vulnerable narcissism (Campbell et al., 2002), as well as dyadic concepts such as emotional responsiveness (Rhodewalt et al., 1998), attachment (Foster et al., 2007), and dysfunctional coping strategies (e.g., alcohol abuse; Bentall et al., 2011). Self-esteem stability is thus related to an individual's general life satisfaction (Oosterwegel et al., 2001) and can be assumed to be associated with satisfaction in interactions, such as dyadic relationship satisfaction, as well.

Despite the relevance and growing interest in self-esteem stability, only a few inventories of the construct are currently available. This might be because previous research on cross-sectional self-esteem stability inventories has shown only small to medium correlations with longitudinal measures of variation in self-esteem (Chabrol et al., 2006). Hence, there is a need to develop new competing measures that can predict outcomes better than what is currently available.

In the present paper, we review the existing self-esteem stability inventories and present a new brief inventory developed on the basis of this review. We report data on construct and criterion validity and compare the new instrument to existing measures.

## Measuring self-esteem stability

There are two general approaches to assessing self-esteem stability. The first is a cross-sectional direct assessment via a scale that is administered once; the second is an indirect assessment in which self-esteem level is measured multiple times, usually with Rosenberg's Self-Esteem Scale (RSES; Rosenberg, 1965), and the standard deviation of the means is calculated (Kernis et al., 1989; Kernis, 2005). The latter is considered to provide the most valid assessment and is hence the “gold standard” (Chabrol et al., 2006, p. 137) against which newly developed scales have to be measured.

Although the latter procedure assesses variability in a naturalistic context, it requires participants to invest considerably more time and effort because they have to fill out the RSES repeatedly and without prompting from the researchers, and then return the questionnaires. These issues might keep researchers from applying this procedure in their studies.

When cross-sectional measures are used, participants are asked to directly rate any fluctuations in self-esteem they tend to experience on a single measurement occasion. Such measures are therefore much more economical. Two of their major limitations, however, are memory bias and validity. First, there is the risk of memory biases since direct assessments require subjects to retrospect over their past experiences which is prone to memory distortion effects (e.g., Schacter, 1999). Second, direct self-esteem stability measures typically only havemedium-sized correlations with the aforementioned “gold standard.” In the following section, we briefly review the existing direct measures.

## Current measures of direct stability

To our knowledge, three cross-sectional measures assessing self-esteem stability with a direct approach are available.

The most recently published of these is the Instability of Self-Esteem Scale (ISES) by Chabrol et al. (2006). Participants are asked to indicate their degree of agreement with the following four items, all of which are very similar in structure and phrasing:

- Item 1: Sometimes I feel worthless; at other times I feel that I am worthwhile.
- Item 2: Sometimes I feel happy with myself; at other times I feel very unhappy with myself.
- Item 3: Sometimes I feel useless; at other times I feel very useful.
- Item 4: Sometimes I feel very bad about myself; at other times I feel very good about myself.

Of course, internal consistency can be expected to be very high due to this extreme degree of overlap.

The scale's developers reported that the correlation between their scale and the SD of repeated assessments using the RSES was 0.81. As this is almost identical to the internal consistency of the ISES, this result suggests that the two measures assess exactly the same construct with equivalent validity, despite their very different approaches. Given that, to our knowledge, other self-esteem stability studies have consistently reported much lower correlations between cross-sectional and longitudinal stability measures (Kernis et al., 1989, 1992; Marsh, 1993; Webster et al., 2017), this result is very surprising.

The second inventory is a derivative of the RSES presented by Kernis et al. (1992). Participants have to estimate “how much they thought they would change their (dis)agreement on a day-to-day basis with each of the items on Rosenberg's Self-Esteem Scale” (p. 628). Of course, this requires participants to have unrealistically high self-reflection abilities. Accordingly, the authors did not find that this measure was significantly correlated with the SD of repeated RSES measurements. Consequently, they evaluated their scale as insufficient, and we did not include it in the present research.

The third and oldest scale is the five-item Stability of Self Scale (RSS) by Rosenberg (1965). Existing research on this scale has shown only weak correlations with longitudinally measured short-term (Kernis et al., 1989, 1992) and long-term self-esteem stability (Marsh, 1993). Only Webster et al. (2017) were able to obtain a moderate mean correlation of 0.31 in a recent meta-analysis. Although they incorporated only eight articles as well as almost 50% unpublished data in their analysis, we agree with the authors that the RSS “deserves a second look” (p. 12) and thus included it in the present research.

In recent decades, scales on state self-esteem (e.g., the State Self-Esteem Scale by Heatherton and Polivy, 1991) have been developed and used to measure temporary fluctuations in self-esteem (Linton and Marriott, 1996). These scales show high similarity with the RSES. First, the item content of current state self-esteem measures is quite similar to the RSES (e.g., “I feel confident about my abilities” from the aforementioned inventory and “I am able to do things as well as most other people” from the RSES). Second, the procedure to assess stability, making the instructions time-bound to the present moment (e.g., “How true are these statements for you RIGHT NOW”) and then having respondents complete the measure repeatedly, is the same as that of Kernis and colleagues, who applied the more prominent RSES with similar changes to the instructions (as described above). Therefore, applying state self-esteem scales is likely to yield comparable results.

From this brief review, we concluded that self-esteem stability is a relevant variable for psychological functioning. However, it is not always possible for researchers to apply the most valid assessment method, presenting the RSES multiple times in order to calculate the standard deviation. Therefore, a reliable and valid direct measurement approach would make an important contribution to improving research on self-esteem stability. A few measures exist but are either insufficient or their psychometric qualities are dubious.

In the present research, we developed a new measure (Study 1) on the basis of the critical issues described above and validated it with relation to individual outcomes (Studies 2 and 3) and dyadic outcomes (Study 4) in comparison with previous measures. To maximize ecological validity, different types of assessments and samples were used: both paper-and-pencil and online assessments, with individuals and couples, and workers and students, who filled out the measure independently at home and in the laboratory. All assessments were anonymous. All analyses were conducted with German samples; therefore, the German versions of the measures were used (see Appendix A2). Data and material of the presented studies are openly accessible at osf.io/sy59r.

## Study 1

The aim of Study 1 was to develop a new, brief scale to directly assess self-esteem stability while overcoming the shortcomings of the existing measures described above. We expected to find unidimensionality and at least satisfactory psychometric properties for the new scale, which we called the Self-Esteem Stability Scale (SESS).

### Method

#### Procedure

We constructed 18 items in German with a balance between positive and negative phrasing. The language style was kept similar to the RSES to enable joint administration. The content of the items was chosen to mirror the major aspects of the RSES (e.g., having a positive attitude toward oneself or having a positive evaluation of one's own abilities compared with others) to ensure content validity. Six of those 18 initial items were chosen (see Supplementary Material) on the basis of a discussion by a group of four personality assessment experts and administered to the sample in an online questionnaire. A 6-point Likert scale (1 = “Does not apply to me” to 6 = “Does apply to me”) was used. Higher scores represent higher reported stability.

#### Sample

Participants were recruited via online postings in social media and via email forwarding. Digital leaflets with information about the study and including a call for volunteers were posted in Facebook groups for students of all fields and in groups for workers in a number of different fields such as healthcare and engineering. Email forwarding was used for people who showed interest in the study but were aware of the potential hypotheses (e.g., psychology students) and therefore had to be excluded. They were asked to email the information about the study to friends and relatives. The total sample was *N* = 215 (70.2% female), aged 18 to 67 (*M* = 32.9, *SD* = 9.0), of which 63% were workers, 32% students, and 5% unemployed or retired. Participants provided informed consent (concerning the purpose of the study, the scientific use of the data, and anonymity) as required by the University of Duisburg-Essen Psychological Institute's Ethics Committee, which approved of the study.

### Results and discussion

#### Dimensionality

Parallel analysis (PA; with 1,000 sets, 95th percentile; see Horn, 1965) of the six items resulted in a one-factor solution (KMO = 0.81; eigenvalues for original data and random PA data for the six possible factors were 2.67/1.23, 0.99/1.11, 0.67/1.03, 0.66/0.95, 0,54/0.87, and 0.44/0.78). Factor analysis was used to exclude items that explained variance of the common latent factor also explained by other items. In contrast to the 0.30 minimal loading often used for larger scales (Costello and Osborne, 2005), we decided to use the much stricter criterion of 0.60 for item inclusion to ensure sufficient internal consistency due to the small number of items on this very brief scale. We thus included three items in the scale (see descriptive statistics Table 1 and factor loadings Table S1), which explained 65.4% of the variance in the factor analysis.

**Table 1:** Psychometric properties of the SESS.

Items^*^	***M***	***SD***	***r*_it_**	***rp***
My attitude toward myself is very stable.	4.11	1.34	0.59	0.73
How I estimate my abilities compared with others changes frequently.	3.86	1.26	0.54	0.69
My positive and negative feelings toward myself often blend into each other.	4.41	1.21	0.55	0.64

*Responses were made on a 6-point Likert scale. M, mean; SD, standard deviation; r_it_, part-whole-corrected item-total correlation; rp, response probability*.

* *English translation*.

#### Psychometric properties

The means and standard deviations of the three SESS items as well as their response probabilities and the part-whole corrected item-total correlations are shown in Table 1. Cronbach's α was 0.73, which is satisfactory considering that Cronbach's α strongly depends on item number. The results indicated that the SESS has at least satisfactory psychometric properties.

## Study 2

The aim of Study 2 was to evaluate the validity of the SESS, developed in Study 1, in comparison to the other aforementioned direct scales (i.e., ISES and RSS).

As argued above, the best evidence of the validity of a direct measure is the extent to which it is able to predict the indirect longitudinal measurement of the same construct. Furthermore, it has been argued that high self-esteem stability should be associated with higher life satisfaction scores as a general indicator of psychological functioning. Therefore, we analyzed the relations between the direct measures and the indirect self-esteem assessment (the “gold standard”) as well as life satisfaction as general indicators of validity.

We expected the new SESS to explain significantly more variance than the ISES or RSS for both the indirect measure of stability and life satisfaction.

### Method

#### Procedure

For our longitudinal measure, we adapted the procedure that has frequently been applied by Kernis et al. (2008). Participants filled out the modified RSES (followed by the Satisfaction with Life Scale) once. They were then given four copies of the modified RSES to fill out on each of the following 2 days at both 10 a.m. and 8 p.m. They were to return these sheets the day after those 2 days, at which point they again received four new copies for the remaining 2 days. Each participant received 10 Euro (approximately $10 US) when all sheets had been returned.

There is a risk that subjects filled out several questionnaires at one time instead of at the specific time as instructed. To reduce this risk, subjects received the copies at three instances (one upon arrival, four thereafter, and four after having returned the previous four). Furthermore, after participants were paid and assured that they can keep the payment, a brief interview was conducted to check the extent to which they had complied with the instructions. Participants who showed any signs of lack of compliance (e.g., deviation from the given time frame to complete the questionnaires or even hesitation to answer) were not included in the analyses.

#### Sample

Participants were recruited on campus with notices and leaflets, as well as via social media postings, e.g., in local Facebook groups (see Study 1). The total sample consisted of 218 participants (75.2% females) between the ages of 18 and 67 (*M* = 28.6, *SD* = 9.4) and was used for the cross-sectional analyses. Of this sample, 64.2% were university students, 30.3 workers, 2.3% school students or apprentices, and 2.3% unemployed (0.9% missing).

Of those 218, a subsample of *N* = 125 returned all RSES questionnaires used in the longitudinal analyses. The latter subsample was significantly younger compared with those who did not return all RSES questionnaires [*t*_(114.8)_ = 11.5, *p* < 0.001]. There were neither associations between the two subsamples with gender [$\chi_{\left(1,\;\text{N}\;=\;218\right)}^{2}$ = 3.58, n.s.], nor with employment status [$\chi_{\left(3,\;\text{N}\;=\;216\right)}^{2}$ = 7.70, n.s.], nor a difference in RSES [*t*_(139.0)_ = 0.572, n.s.]. A difference in SESS occurred [*t*_(216)_ = 4.01, *p* < 0.001] with a higher mean level of instability among those who returned all questionnaires.

Participants provided informed consent (concerning the purpose of the study, the scientific use of the data, and anonymity) as required by University of Duisburg-Essen Psychological Institute's Ethics Committee, which approved of the study.

#### Measures

##### Self-esteem stability

Self-esteem stability was measured in two ways: indirectly via a longitudinal design and directly via a cross-sectional design. In the indirect assessment, participants completed a modified version of the RSES (Rosenberg, 1965) over a period of 5 days (see section Procedure). For this purpose, the RSES instructions were modified so that participants were asked to indicate how they felt about themselves in the present moment, rather than in general (see Kernis et al., 2008; present sample α = 0.91). The adapted instructions read: “Please indicate to what extent the following statements apply to you personally RIGHT NOW in the present moment.” Item containing a general reference (e.g., “I certainly feel useless at times”) were rephrased by omitting the general reference “at times.” The standard deviation was calculated to assess the extent of short-term fluctuations in self-esteem.

We applied the SESS (α = 0.71) developed in Study 1, the ISES (α = 0.91), and the RSS (α = 0.87), described in detail above, as direct assessments.

##### Life satisfaction

Life satisfaction was assessed once using the Satisfaction with Life Scale (SWLS) developed by Diener et al. (1985). The SWLS is a five-item instrument designed to measure one's global cognitive judgment of one's life. In this study, an internal consistency of α = 0.81 was obtained.

German versions of all measures were used (see Appendix).

### Results and discussion

#### Criterion validity: relation to the longitudinal indirect assessment of stability of self-esteem

To compare the ability of the different direct measures of self-esteem stability (SESS, ISES, RSS) to predict the longitudinal indirect assessment using the RSES, we conducted a multiple regression analysis in which the predictors were entered in one step. As shown in Table 2, the best predictor of the longitudinal assessment of self-esteem was the SESS, whereas neither the ISES nor the RSS were incrementally predictive. Table 2 also includes the partial correlations between the criterion variable and each of the predictors, controlling for the two remaining predictors. These indices again demonstrated that only the SESS was substantially associated with the longitudinal assessment above and beyond the other two direct measures. All associations were as assumed: higher scores in stability were associated with lower instability.

**Table 2:** Multiple regression analyses predicting the indirect measure of self-esteem stability assessed by multiple RSES administrations and satisfaction with life.

	**Self-esteem instability (*****SD*** **of multiple RSES assessments)**				**Satisfaction with life (SWLS)**			
**Variable**	**B**	**SE**	**B**	***r*_pc_**	**B**	**SE**	**β**	***r*_pc_**
SESS	−0.11	0.05	−0.11^*^	−0.20^*^	0.35	0.08	0.51^***^	0.35^***^
ISES	0.05	0.04	0.05	0.11	−0.11	0.07	−0.18	−0.13
RSS	0.01	0.04	0.01	0.02	0.15	0.07	0.24^*^	0.17^*^
	*R*^2^ =	0.17^***^			*R*^2^ =	0.21^***^		

*SWLS, Satisfaction With Life Scale; SESS, Stability of Self-Esteem Scale; ISES, Instability of Self-Esteem Scale; RSS, Rosenberg Stability of Self Scale; r_pc_, partial correlation between predictor and criterion controlling for the other predictors, respectively. Correlations of self-esteem instability with direct measures were for SESS r = −0.40, for ISES r = 0.36, and for RSS r = −0.30, respectively, all p < 0.001*.

* p ≤ 0.05;

** p ≤ 0.01;

*** *p ≤ 0.001*.

It is worth mentioning here that all three indirect measures were significantly correlated with one another, yet only moderately correlated with the variability assessed longitudinally via multiple RSES measurements (see Table 2; note: complete correlation table see Supplementary Material). There are at least two possible interpretations of this: as calling the validity of the direct measurement approach as such into question, or more likely, suggesting that the direct measures assess a specific self-reflective aspect of self-esteem stability, which can be described as an individual's self-concept related to their experience of self-evaluative stability. This issue will be considered in the Discussion section.

#### Construct validity: prediction of life satisfaction

We applied the same analysis strategy as described above to predict life satisfaction using the three direct stability measures. Table 2 again shows that of the three measures, the SESS proved to be the best predictor of higher life satisfaction. In this instance, the RSS explained additional significant variance. The SESS also exhibited the strongest positive relationship with life satisfaction in the partial correlations that controlled for the other predictors.

In sum, the SESS exhibited greater predictive power than the ISES or RSS with respect to both the “gold standard” of self-esteem stability and life satisfaction as a general indicator of psychological functioning. However, the predictive power of all measures was small to medium, in line with previous research (Kernis et al., 1989; Oosterwegel et al., 2001; Webster et al., 2017).

## Study 3

The first aim of Study 3 was to further analyze the validity of the SESS in comparison to the two other scales directly assessing self-esteem stability (ISES and RSS, see above). For this purpose, we chose neuroticism and vulnerable narcissism as two personality traits that are defined by a high level of instability. Whereas instability in emotional states in general is characteristic of high neuroticism (Costa and McCrae, 1992), instability of self-image is characteristic of high levels of vulnerable narcissism (Rhodewalt et al., 1998). Therefore, we viewed both traits as appropriate criteria for validity.

Previous studies have reported substantial correlations between stability measures and the general level of self-esteem (Kernis and Grannemann, 1991; Marsh, 1993; Roberts et al., 1995; Okada, 2010). A valid stability measure would have to substantially predict the outcome variables over and above the predictive power of the level of self-esteem. Therefore, the study's second aim was to evaluate the incremental validity of the three stability measures in predicting the aforementioned criteria over and above general level of self-esteem.

### Method

#### Sample

Participants were recruited on campus with notices and leaflets as well as via social media postings, e.g., in local Facebook groups (see section Study 1). The total sample consisted of 287 students (77.7% female) between the ages of 18 and 38 (*M* = 22.6, *SD* = 3.3). Participants provided informed consent (concerning the purpose of the study, the scientific use of the data, and anonymity) as required by the University of Duisburg-Essen Psychological Institute's Ethics Committee, which approved of the study.

#### Measures

##### Self-esteem level

Self-esteem was assessed with the RSES (Rosenberg, 1965) as a global self-esteem measure. The 10-item RSES is conceptualized as a single-factor scale and is considered to be unidimensional (Roth et al., 2008). The internal consistency in the present sample was α = 0.88.

##### Self-esteem stability

We applied the SESS (α = 0.76) from Study 1, as well as the ISES (α = 0.89) and the RSS (α = 0.86), described above, as direct assessments.

##### Neuroticism

We used the Neuroticism subscale of the NEO Five Factor Inventory (NEO-FFI) by Costa and McCrae (1992). This subscale consists of 12 items in a 5-point Likert scale format. The reliability in the present study was α = 0.81.

##### Vulnerable narcissism

The Vulnerability subscale of the Brief-Pathological Narcissism Inventory (B-PNI; Schoenleber et al., 2015), an established brief version of the Pathological Narcissism Inventory (PNI; Pincus et al., 2009), was used as a measure of vulnerable narcissism. This subscale consists of 16 items. Cronbach's alpha was α = 0.87.

As before, German versions of the scales were used (see Appendix).

### Results and discussion

We chose the same analysis strategy as described in Study 2. Regression analyses were conducted with the three direct stability measures (SESS, ISES, and RSS) as predictors (note: complete correlation table see Supplementary Material). All predictors were included in the model in a single step to identify the best predictor of the criterion variables neuroticism and vulnerable narcissism.

As shown in Table 3, the SESS turned out to be the best predictor of both neuroticism and vulnerable narcissism, but the ISES and RSS were also significant predictors. All associations were as assumed: higher scores on stability were associated with lower neuroticism and narcissism. The SESS exhibited the highest substantial relations to both criteria, as indicated by the partial correlations between each of the predictors and the criteria, controlling for the remaining two predictors.

**Table 3:** Multiple regression analyses in predicting neuroticism and vulnerable narcissism.

	**Neuroticism (NEO-FFI N)**				**Vulnerable narcissism (B-PNI V)**			
**Variable**	**B**	**SE**	**β**	***r*_pc_**	**B**	**SE**	**β**	***r*_pc_**
SESS	−0.26	0.05	−0.34^***^	−0.21^***^	−0.19	0.05	−0.27^***^	−0.28^***^
ISES	0.12	0.04	0.18^*^	0.15^*^	0.10	0.04	0.18^*^	0.17^**^
RSS	−0.13	0.05	−0.18^**^	−0.11	−0.10	0.05	−0.16	−0.14^*^
	*R*^2^ =	0.17^***^			*R*^2^ =	0.28^***^		

*NEO-FFI N NEO, Five Factor Inventory; B-PNI V, Brief-Pathological Narcissism Inventory Vulnerability; SESS, Stability of Self-Esteem Scale; ISES, Instability of Self-Esteem Scale; RSS, Rosenberg Stability of Self Scale; r_pc_, partial correlation between predictor and criterion controlling for the other two predictor variables, respectively*.

* p ≤ 0.05;

** p ≤ 0.01;

*** *p ≤ 0.001*.

Because the direct stability measures were highly correlated with the general level of self-esteem (SESS: *r* = 0.55, ISES: *r* = −0.58, RSS: *r* = 0.62; all *p*s < 0.001), we analyzed the extent to which the three stability measures exhibited incremental validity over and above the general level of self-esteem in predicting neuroticism and vulnerable narcissism. For this purpose, hierarchical two-step regression models were calculated in which the general level of self-esteem was entered as a predictor in the first step, and the three stability measures were entered in the second step.

As shown in Table 4, all three stability measures exhibited small but significant incremental validity over the general level of self-esteem, as indicated by the Δ*R*^2^ coefficients. Differences between the direct measures were small; however, the SESS appeared to be the strongest predictor of the three.

**Table 4:** Results of multiple regression analyses predicting neuroticism and vulnerable narcissism.

	**Step 1** ***R***^**2**^		**Step 2** Δ***R***^**2**^	
**Criterion**	**Models 1-3RSES**	**Model 1SESS**	**Model 2ISES**	**Model 3RSS**
Neuroticism (NEO-FFI N)	0.41^***^	0.07^***^	0.03^***^	0.04^***^
Vulnerable narcissism (B-PNI V)	0.28^***^	0.06^***^	0.03^***^	0.03^***^

*NEO-FFI N NEO, Five Factor Inventory; B-PNI V, Brief-Pathological Narcissism Inventory Vulnerability; RSES, Rosenberg's Self-Esteem Scale (general level of self-esteem); SESS, Stability of Self-Esteem Scale; ISES, Instability of Self-Esteem Scale; RSS, Stability of Self Scale*.

*** *p ≤ .001*.

## Study 4

Whereas, Studies 2 and 3 analyzed the validity of the SESS with regard to individual outcomes (e.g., life satisfaction), Study 4 focused on validity with regard to a dyadic outcome. The aim of Study 4 was to evaluate the validity of our three-item SESS with respect to predicting couples' relationship satisfaction. As mentioned above, the perception that one's partner has unstable self-esteem seems to be a crucial issue for low relationship satisfaction.

As shown by Altmann et al. (2013) in a study of couples, relationship satisfaction primarily depends on how people view their partner, not on how they view themselves. Therefore, we expected that the rating of one's partner's self-esteem stability would be the most relevant predictor of relationship satisfaction. Because of the high correlations between self-esteem and stability of self-esteem, the incremental validity of the stability measure over general self-esteem was once again considered in the analyses.

### Method

#### Procedure

Participants were recruited in local institutions such as schools (teachers), sports clubs, on campus (academic and nonacademic personnel as well as students), and via social media postings, e.g., in local Facebook groups (see Study 1). Interested persons received paper-and-pencil questionnaires with two stamped return envelopes for themselves and their partner if both were of legal age and both would describe themselves as being in an ongoing, stable, long-term relationship. Anonymity concerns by several potential participants forced us to omit questions concerning their current work situation.

#### Sample

The sample consisted of 106 participants comprising 53 heterosexual couples between 18 and 80 years old (*M* = 35.2, *SD* = 16.0). Of the sample, 49.7% had a university entrance qualification (Abitur), 40.3% had a university degree, and 10.0% had a lower secondary degree or no degree so far. The length of relationship ranged from 0.7 to 58 years (*M* = 12.2, *SD* = 13.6), 32.1% of the participants were married, and 61.3% were living together. Participants provided informed consent (concerning the purpose of the study, the scientific use of the data, and anonymity) as required by the University of Duisburg-Essen Psychological Institute's Ethics Committee, which approved of the study.

#### Measures

##### Self-ratings

We measured the global level of self-esteem with the RSES (Rosenberg, 1965) as described above. Self-esteem stability was directly assessed with the three-item SESS developed in Study 1. The Relationship Assessment Scale (RAS; Hendrick, 1988) was used to evaluate relationship satisfaction. The RAS consists of seven items and was designed to be a general measure of relationship satisfaction. Sample items include “How well does your partner meet your needs?” and “In general, how satisfied are you with your relationship?” Items were rated on a 7-point Likert scale, with larger values indicating higher levels of satisfaction.

##### Other ratings

Partner ratings were also gathered with regard to the global level of self-esteem and self-esteem stability using specially created partner versions of the RSES and SESS. In these modified versions, participants were asked to indicate the extent to which they agreed with statements describing their relationship partner. All RSES and SESS items were reworded by replacing first-person language with third-person language. For example, “How I estimate my abilities compared with others changes frequently” was changed to “How my partner estimates his/her abilities compared with others changes frequently.”

As before, we used German versions of all measures (see Appendix).

### Results and discussion

#### Interrater agreement

Agreement about one another's self-esteem as well as the stability of self-esteem (i.e., the correlations between self-ratings and other ratings) were analyzed with intraclass correlations (ICC). Interrater agreement was ICC = 0.42 (*p* < 0.001) for level of self-esteem and ICC = 0.41 (*p* < 0.001) for self-esteem stability. These results are comparable to previous findings on self-other agreement regarding personality in samples of couples (Watson et al., 2000a,b). A meta-analysis of 28 studies of romantic partners by Fletcher and Kerr (2010) revealed a mean effect size for tracking accuracy in personality traits of 0.43.

#### Relation between relationship satisfaction and self-esteem

To compare the predictive power of self-esteem stability as measured by the SESS to the predictive power of the general level of self-esteem as measured by the RSES, relationship satisfaction was predicted with two regression models. In the first regression model, the RSES was entered in a first step, followed by SESS in a second step, and vice versa in the second model. Both models were calculated for self-ratings, other ratings of the same person, and that person's other ratings of their partner (see Table 5; note: complete correlation table see Supplementary Material).

**Table 5:** Head-to-Head Comparisons of the RSES and SESS in the Concurrent Prediction of Relationship Satisfaction (RAS) in a Multiple Regression Analysis.

**Models/Steps**	**Self-ratings**	**Other ratings of target by partner**	**Other ratings of partner by target**
**Model 1**			
Step 1: *R*2 (RSES)	0.02^*^	0.00	0.07^**^
Step 2: Δ*R*2 (SESS)	0.04^*^	0.01	0.04^*^
**Model 2**			
Step 1: *R*2 (SESS)	0.06^*^	0.01	0.11^***^
Step 2: Δ*R*2 (RSES)	0.00	0.00	0.00
Total *R*2	0.06^*^	0.01	0.11^**^

* p ≤ 0.05;

** p ≤ 0.01;

*** *p ≤ 0.001*.

As expected, head-to-head comparisons revealed that ratings of one's partner were the best predictors of relationship satisfaction, followed by self-ratings. By contrast, other ratings by partners did not predict relationship satisfaction. It is interesting that there was clear evidence for the incremental validity of the SESS as a stability measure over and above the general level of self-esteem, whereas general-self-esteem did not contribute incrementally to predicting relationship satisfaction over and above the SESS.

This provides clear evidence for the relevance of the SESS, which demonstrated predictive power beyond the general level of self-esteem. The results are even more relevant in light of the fact that relationship satisfaction exhibited ceiling effects (moderately skewed with *v* = 0.87, *M* = 4.3, *SD* = 0.52 on a 5-point Likert scale), meaning that the variance was limited and significant results were thus unlikely. All associations were as assumed: higher self-esteem level and stability were associated with higher relationship satisfaction.

## General discussion

Self-esteem stability is considered an important variable for psychological functioning and is receiving growing attention. The few inventories involving direct cross-sectional assessment exhibit similar deficits in validity, making new developments necessary. The newly developed Self-esteem Stability Scale (SESS), presented here in Study 1 and validated in Studies 2 to 4, represents such a new measure. Psychometric properties were found to be satisfactory, and the factor structure was unidimensional, as expected. The SESS was found to have superior predictive power compared to existing measures and demonstrated incremental validity beyond self-esteem level.

The fact that previous studies and the results presented here have consistently shown that self-esteem stability is incrementally predictive over self-esteem level further supports the notion that both of these aspects should be taken into account when operationalizing self-esteem. The joint administration of very brief SESS, which encompasses only three items, with the RSES is economical, relatively efficient, and exhibits significant incremental predictive power above and beyond the RSES with regard to both individual and dyadic outcomes.

### Measuring self-esteem stability

Our analyses showed that all direct self-esteem stability measures were substantially correlated with the “gold standard” (i.e., indirect, longitudinally assessed variability). However, even the SESS, the best predictor, yielded only moderate results. Thus, the indirect and direct measures might assess different aspects ofself-esteemstability, which makes sense given their quite different requirements.

The indirect assessment requires participants to rate their self-esteem level “at the present moment” at several different times points, and therefore presumably assesses actualself-esteemvariability. One has to bear in mind, however, that the questionnaires are filled out by participants on their own accord, at specific moments in their lives, and only when the participant is willing to do so in a particular situation. It is highly unlikely that participants will pause, sit down, and fill out the RSES when they are in a moment of flow or ecstasy, or in an acute self-esteem threatening situation. This measurement approach might therefore work best when the research question focuses on non-extreme or normal day-to-day variation, or when one's ability to self-reflect is reduced (e.g., people with depression, of whom realistic retrospection cannot be expected).

The direct assessment, on the other hand, requires participants to reflect on their general experiences of fluctuation in their self-evaluations. Therefore, this approach might primarily assess a retrospective construct, namely one's individual self-concept of one's stability of self-evaluations, i.e., the aggregate of the self-esteem fluctuations experienced in the weeks and months prior to the assessment. The results of our study further indicate that this might be influenced by a person's general self-image of their general emotional stability, as we found substantial and significant correlations with neuroticism and vulnerable narcissism. This measurement approach might function best for research on the general population and with subjects with an intact self-reflective ability.

### Further developments

Self-esteem stability scales are still in the process of optimization and still have challenges to overcome. We will discuss two such challenges in detail here.

First, further research is needed on the definition of the time period to which self-esteem stability refers. There is not yet a standard on what number of assessments or period of time is optimal for providing the best estimate of short-term variations when using the indirect assessment. It seems beneficial for future research to distinguish between variability that occurs within a day compared to that which occurs over a period of 2–4 weeks, as they might have vastly different causes and consequences. Daily, weekly, and monthly changes might be influenced by different factors and might therefore produce different incremental values. Examining the relationships between contingent self-esteem (e.g., Crocker and Wolfe, 2001), short-term fluctuations and personality development, and long-term stability in self-esteem (e.g., Trzesniewski et al., 2003) might offer a beneficial structure.

The second issue is the generally high correlation with self-esteem level. Subjects might have trouble answering self-esteem items asking them to reflect only on the present moment. Their general self-esteem level might be more accessible and might therefore influence or distort their response. Implicit measurements could be a solution if there is no substantial discrepancy between the subjects explicit and implicit self-esteem. Further research might use scenarios or items with symbols (e.g., asking subjects to indicate which of the presented body postures reflects how they feel about themselves right now) to make it easier for respondents to express their current feelings and evaluations of themselves.

### Limitations

The results presented here must naturally be treated with caution. The samples tended to be predominantly female and were recruited on a voluntary basis, which might have produced a bias that cannot be discounted due to the non-representativeness of the sample.

The risk of subjects filling out several self-esteem questionnaires at once instead of at different times as instructed remains. Comparably, all questionnaire data rest upon the assumption that subjects are honest and conscientious instead of providing random answers. Even handing out only one copy at a time cannot guarantee honest participation. In the present studies, questionnaires we kept extremely short, interviewed the participants afterwards, and paid comparably well to further minimize the likelihood of deception.

Furthermore, we administered the German versions of all inventories to German-speaking samples. Thus, the generalizability of our results to other populations (particularly those from different cultural circles such as Asia) and their replicability in different languages cannot be guaranteed and must be the subject of further studies. Given that the items of the newly presented SESS were phrased similarly as previous stability measures as well as the Rosenberg Self-esteem Scale, the likelihood of achieving comparable results in English-speaking populations can be considered to be rather high. We hope to stimulate further research on self-esteem stability by providing the items of the SESS in English and German.

## Author contributions

TA and MR: Substantial contributions to the conception or design of the work, the acquisition, analysis, and interpretation of data for the work. TA and MR: Drafting the work and revising it critically for important intellectual content. TA and MR: Final approval of the version to be published. TA and MR: Agreement to be accountable for all aspects of the work in ensuring that questions related to the accuracy or integrity of any part of the work are appropriately investigated and resolved.

### Conflict of interest statement

The authors declare that the research was conducted in the absence of any commercial or financial relationships that could be construed as a potential conflict of interest.
