# Supplementary material for: The Self-esteem Stability Scale (SESS) for Cross-Sectional Direct Assessment of Self-esteem Stability
Source: Front Psychol. 2018 Feb 13;9:91. doi: 10.3389/fpsyg.2018.00091 (PMC5816969; doi:10.3389/fpsyg.2018.00091)
Supplement: Supplementary file 1 [file DataSheet1.pdf]

## Supplementary Material

**S1 Initial item pool**
**S2 Intercorrelation tables for Study 2-4**
**S3 Means and standard deviations for Study 2-4**

### S1 Initial item pool

English translation in parentheses.

| # | Item | Reason for exclusion |
|---|---|---|
| Items included in the final version of the SESS | | |
| 01 | Meine Einstellung zu mir selbst ist sehr stabil.<br>(My attitude toward myself is very stable.) | (included based on factor loading = .73) |
| 02 | Wie ich meine eigenen Fähigkeiten im Vergleich zu anderen einschätze, wechselt häufig.<br>(How I estimate my abilities compared with others changes frequently.) | (included based on factor loading = .72) |
| 03 | Positive und negative Gefühle mir selbst gegenüber gehen oft ineinander über.<br>(My positive and negative feelings toward myself often blend into each other.) | (included based on factor loading = .64) |
| Items excluded empirically in Study 1 | | |
| 04 | Meine Meinung über mich selbst neigt dazu sich zu ändern statt immer gleich zu bleiben.<br>(My attitude about myself tends to change instead of always remaining the same.) | Excluded empirically in Study 1 (factor loading .58 < .60) |
| 05 | Die Achtung, die ich vor mir selbst habe, schwankt über die Zeit hinweg nur sehr wenig.<br>(The respect I have for myself fluctuates only slightly over time.) | Excluded empirically in Study 1 (factor loading .10 < .60) |
| 06 | Wie sehr ich mit mir zufrieden bin, schwankt von Zeit zu Zeit.<br>(My satisfaction with myself fluctuates from time to time.) | Excluded empirically in Study 1 (factor loading .58 < .60) |
| Items excluded prior to empirical scale development | | |
| 07 | Dass sich die Meinung ändert, die ich von mir selbst habe, ist sehr unwahrscheinlich.<br>(Changes in the opinion I have about myself are unlikely.) | Wording too complicated |
| 08 | Die Achtung, die ich vor mir selbst habe, verändert sich von Zeit zu Zeit.<br>(The respect I have for myself changes from time to time.) | More than one plausible interpretation of item content |
| 09 | Die Art, wie ich meine eigenen Fähigkeiten einschätze, wechselt häufig.<br>(The way I estimate my abilities changes often.) | More than one plausible interpretation of item content |
| 10 | Meine Einstellungen zu mir selbst sind sehr unbeständig/ nicht sehr stabil.<br>(My attitudes about myself are volatile / not very stable.) | Wording outdated |
| 11 | Meine Meinung über mich selbst ist sehr stabil / immer gleich.<br>(My opinion about myself is very stable / always the same.) | More than one plausible interpretation of item content |
| 12 | Meine Meinung über mich selbst neigt dazu, sich erheblich zu ändern statt immer gleich zu bleiben.<br>(My opinion about myself tends to changes considerably instead of always remaining the same.) | Wording too intense |

| | | |
|---|---|---|
| 13 | Meine Zufriedenheit mit mir selbst schwankt stark.<br>(My satisfaction with myself fluctuates intensely.) | Wording too intense |
| 14 | Meine Zufriedenheit mit mir selbst schwankt.<br>(My satisfaction with myself fluctuates.) | Uncommon phrasing |
| 15 | Meine Zufriedenheit mit mir selbst ist sehr stabil.<br>(My satisfaction with myself is very stable.) | Wording too intense |
| 16 | Ob ich mir selbst gegenüber positive und negative Gefühle habe, wechselt häufig.<br>(Whether I have positive or negative feelings about myself changes often.) | Wording too complicated |
| 17 | Positive und negative Gefühle gehen oft ineinander über / wechseln sich rasch ab.<br>(positive and negative feelings often blend into each other / alternate quickly.) | Two items in one |
| 18 | Wie sehr ich von mir selbst überzeugt bin oder an mir zweifle ist nicht immer gleich, sondern unterscheidet sich von Situation zu Situation.<br>(How much I am convinced about myself or have doubts about myself is not constant, but differs highly from situation to situation.) | Wording too complicated |

Final version of the SESS

English version

1. My attitude toward myself is very stable.
2. How I estimate my abilities compared with others changes frequently.
3. My positive and negative feelings toward myself often blend into each other.

German version:

1. Meine Einstellung zu mir selbst ist sehr stabil.
2. Wie ich meine eigenen Fähigkeiten im Vergleich zu anderen einschätze, wechselt häufig.
3. Positive und negative Gefühle mir selbst gegenüber gehen oft ineinander über.

**S2 Intercorrelation tables for Study 2-4**

Intercorrelation Table for Study 2

| | SD RSES rep | SESS | ISES | RSS |
|---|---|---|---|---|
| SD RSES rep | 1 | | | |
| SESS | -.40** | 1 | | |
| ISES | .36** | -.70** | 1 | |
| RSS | -.30** | .71** | -.72** | 1 |
| SWLS | -.19* | .46** | -.36** | .25** |

*Note*. SD RSES rep = standard deviation of repeated administrations of the Rosenberg Self-Esteem Scale; SESS = Stability of Self-Esteem Scale; ISES = Instability of Self-Esteem Scale; RSS = Rosenberg Stability of Self Scale; SWLS = Satisfaction With Life Scale
* $p \leq .05$. ** $p \leq .01$.

Intercorrelation Table for Study 3

| | RSES | SESS | ISES | RSS | NEO-FFI N |
|---|---|---|---|---|---|
| RSES | 1 | | | | |
| SESS | .55** | 1 | | | |
| ISES | -.58** | -.59** | 1 | | |
| RSS | .62** | .72** | -.68** | 1 | |
| NEO-FFI N | -.64** | -.58** | .51** | -.55** | 1 |
| B-PNI V | -.53** | -.48** | .44** | -.47** | .56** |

*Note*. RSES = Rosenberg Self-Esteem Scale; SESS = Stability of Self-Esteem Scale; ISES = Instability of Self-Esteem Scale; RSS = Rosenberg Stability of Self Scale; NEO-FFI N NEO = Five Factor Inventory; B-PNI V = Brief-Pathological Narcissism Inventory Vulnerability
** $p \leq .01$.

Intercorrelation Table for Study 4

| | RSES SR | SESS SR | RSES OR | SESS OR |
|---|---|---|---|---|
| RSES SR | 1 | | | |
| SESS SR | .65** | 1 | | |
| RSES OR | .39** | .19 | 1 | |
| SESS OR | .39** | .33** | .74** | 1 |
| RAS SR | .15 | .22* | .27** | .31** |

*Note*. RSES = Rosenberg Self-Esteem Scale; SESS = Stability of Self-Esteem Scale; RAS = Relationship Assessment Scale; SR = self-rating; OR = other rating
* $p \leq .05$. ** $p \leq .01$.

**S3 Means and standard deviations for Study 2-4**

Means and Standard Deviations for Study 2

| Measure | M | SD |
|---|---|---|
| SD RSES rep | 1.15 | 0.38 |
| SESS | 3.87 | 1.11 |
| ISES | 3.58 | 1.27 |
| RSS | 3.69 | 1.17 |
| SWLS | 3.65 | 0.73 |

*Note*. SD RSES rep = standard deviation of repeated administrations of the Rosenberg Self-Esteem Scale; SESS = Stability of Self-Esteem Scale; ISES = Instability of Self-Esteem Scale; RSS = Rosenberg Stability of Self Scale; SWLS = Satisfaction With Life Scale.

Means and Standard Deviations for Study 3

| Measure | M | SD |
|---|---|---|
| RSES | 4.49 | 0.88 |
| SESS | 3.56 | 0.98 |
| ISES | 3.50 | 1.26 |
| RSS | 3.67 | 1.12 |
| NEO-FFI N | 2.91 | 0.77 |
| B-PNI V | 2.55 | 0.72 |

*Note*. RSES = Rosenberg Self-Esteem Scale; SESS = Stability of Self-Esteem Scale; ISES = Instability of Self-Esteem Scale; RSS = Rosenberg Stability of Self Scale; NEO-FFI N NEO = Five Factor Inventory; B-PNI V = Brief-Pathological Narcissism Inventory Vulnerability.

Means and Standard Deviations for Study 4

| Measure | M | SD |
|---|---|---|
| RSES SR | 5.03 | 0.66 |
| SESS SR | 4.10 | 0.85 |
| RSES OR | 4.82 | 0.75 |
| SESS OR | 4.04 | 0.87 |
| RAS SR | 4.35 | 0.52 |

*Note*. RSES = Rosenberg Self-Esteem Scale; SESS = Stability of Self-Esteem Scale; RAS = Relationship Assessment Scale; SR = self-rating; OR = other rating.
