# Supplementary material for: The Self-esteem Stability Scale (SESS) for Cross-Sectional Direct Assessment of Self-esteem Stability
Source: Front Psychol. 2018 Feb 13;9:91. doi: 10.3389/fpsyg.2018.00091 (PMC5816969; doi:10.3389/fpsyg.2018.00091)
Supplement: Supplementary file 2 [file DataSheet2.docx]

**Appendix**

**German versions of the inventories**

as applied in Studies 1-4 in alphabetical order

- Rosenberg Self-Esteem Scale (RSES): Ferring and Filipp (1996)
- Subscale “Neuroticism” of the NEO Five Factor Inventory (NEO-FFI N): Borkenau and Ostendorf (2002)
- Brief-Pathological Narcissism Inventory (B-PNI): Morf et al. (2016)
- Relationship Assessment Scale (RAS): Sander and Böcker (1993)
- Satisfaction With Life Scale (SWLS): Schumacher (2003)
- Instability of Self-Esteem Scale (ISES): translation by bilingual German native speaker, back-translated by bilingual English native speaker, original and back-translation were identical
- Stability of Self Scale (RSS): translation by bilingual German native speaker, back-translated by bilingual English native speaker, original and back-translation were identical

Borkenau, P., & Ostendorf, F. (2002). *NEO-Fünf-Faktoren Inventar nach Costa und McCrae (NEO-FFI).* Göttingen, Germany: Hogrefe.

Ferring, D., & Filipp, S.-H. (1996). Messung des Selbstwertgefühls: Befunde zu Reliabilität, Validität und Stabilität der Rosenberg-Skala [Measurement of self-esteem: Reliability, validity, and stability of the Rosenberg Scale]. *Diagnostica, 42*(3), 284–292.

Morf, C. C., Schürch, E., Küfner, A., Siegrist, P., Vater, A., Back, M., . . . Schröder-Abé, M. (2016). Expanding the nomological net of the Pathological Narcissism Inventory: German validation and extension in a clinical inpatient sample. *Assessment*, Advance online publication. doi:10.1177/1073191115627010

Sander, J., & Böcker, S. (1993). Die Deutsche Form der Relationship Assesment Scale (RAS): Eine kurze Skala zur Messung der Zufriedenheit in einer Partnerschaft. *Diagnostica, 39*, 55-62.

Schumacher, J. (2003). SWLS – Satisfaction with Life Scale. In J. Schumacher, A. Klaiberg, & E. Braehler (Eds.), *Diagnostische Verfahren zu Lebensqualität und Wohlbefinden* *[Diagnostic instruments on quality of life and well-being]* (pp. 305–309). Goettingen, Germany: Hogrefe.
